# Supplementary material for: Assays for Monitoring Apixaban and Rivaroxaban in Emergency Settings, State-of-the-Art Routine Analysis, and Volumetric Absorptive Microsamples Deliver Discordant Results
Source: Diagnostics (Basel). 2024 Sep 2;14(17):1939. doi: 10.3390/diagnostics14171939 (PMC11394325; doi:10.3390/diagnostics14171939)
Supplement: Supplementary file 1 [file diagnostics-14-01939-s001.zip › diagnostics-3119035-supplementary.pdf]

diagnostics

Supplementary Materials

Assays for Monitoring Apixaban and Rivaroxaban in Emergency Settings, State-of-the-Art Routine Analysis, and Volumetric Absorptive Microsamples Deliver Discordant Results

Adrienne Fehér [1,†], István Vincze [1,†], James Rudge [2], Gyula Domján [3], Barna Vásárhelyi [1] and Gellért Balázs Karvaly [1,*]

[1] Department of Laboratory Medicine, Semmelweis University, H-1089 Budapest, Hungary; feher.adrienne@semmelweis.hu (A.F.), vincze.istvan@semmelweis.hu (I.V.), vasarhelyi.barna@semmelweis.hu (B.V.)
[2] Trajan Scientific & Medical, Milton Keynes MK8 0AB, UK; jrudge@trajanscimed.com
[3] Department of Internal Medicine and Oncology, Semmelweis University, H-1083 Budapest, Hungary; domjan.gyula@semmelweis.hu
* Correspondence: karvaly.gellert.balazs@semmelweis.hu
† These authors contributed equally to this work and share first authorship.

A: Fehér and I. Vincze have contributed equally and share first authorship.

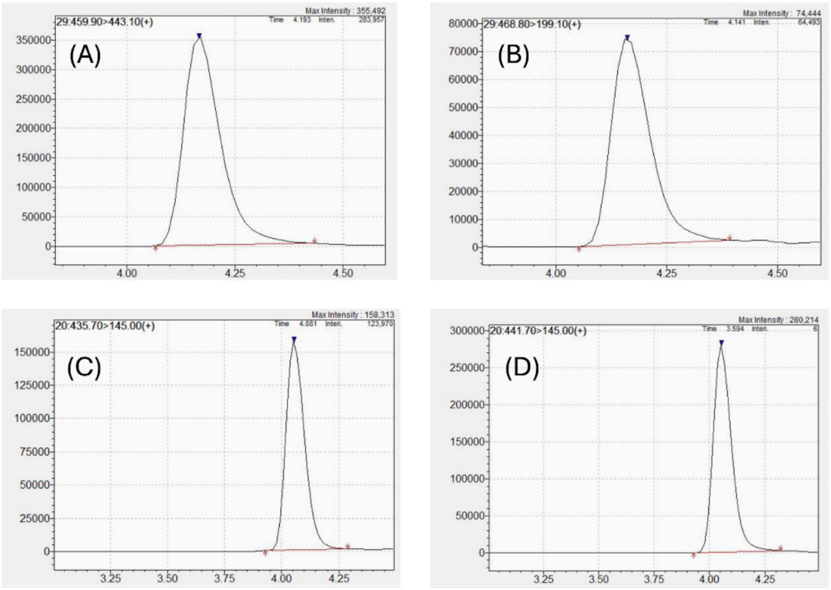

Figure S1. Ion chromatograms of apixaban, rivaroxaban, and their respective internal standards, obtained after processing a liquid plasma calibrator. (A) Apixaban, (B) ^{13}C, $^{2}H_8$-apixaban, (C) rivaroxaban, (D) $^{13}C_6$-rivaroxaban.

Table S1. Hematocrit values, apixaban relative recoveries, as well as the slopes and determination coefficients calculated for each whole blood sample (A thru J) in the investigation of the relationship between hematocrit, analyte concentration, and relative recoveries. The experiments were performed by spiking the analytes to each sample at a low, and at a high level.

Spiking Level	Sample	Hematocrit	Relative Recovery (%)	Slope	Determination Coefficient (r^2)
Low	A	0.286	0.9825	-1.1452	0.945
		0.410	0.9237		
		0.607	0.6263		
	B	0.279	1.0079	-0.9933	0.996
		0.400	0.868698		
		0.593	0.6937		
	C	0.293	0.9829	-0.5328	0.996
		0.420	0.9054		
		0.622	0.8061		
	D	0.300	0.9987	-0.8801	0.933
		0.430	0.9585		
		0.637	0.7126		
	E	0.321	1.0053	-1.1040	0.971
		0.460	0.9163		
		0.681	0.6164		
	F	0.347	1.0453	-1.0737	0.996
		0.533	0.8312		
		0.610	0.7674		
	G	0.339	0.9833	-0.6026	0.924
		0.520	0.9074		
		0.596	0.8181		
	H	0.356	0.8805	-0.2269	0.507
		0.546	0.8824		
		0.625	0.8050		
	I	0.347	1.0080	-0.4577	0.510
		0.533	0.8349		
		0.610	0.9156		
	J	0.356	0.8428	-0.0806	0.051
		0.546	0.7574		
		0.625	0.8433		
High	A	0.286	1.0181	-1.1394	0.957
		0.410	0.9493		
		0.607	0.6622		
	B	0.279	0.9842	-1.2905	0.825
		0.400	1.0020		
		0.593	0.6042		
	C	0.293	0.9983	-1.1212	0.991
		0.420	0.8877		
		0.622	0.6337		
	D	0.300	1.0489	-1.1538	0.999
		0.430	0.9082		
		0.637	0.6614		
	E	0.321	1.0051	-1.2533	0.983

		0.460	0.8868		
		0.681	0.5612		
	F	0.347	0.9106	-0.7282	0.472
		0.533	0.6241		
		0.610	0.7671		
	G	0.339	0.8730	-0.8224	0.979
		0.520	0.7013		
		0.596	0.6692		
	H	0.356	0.8529	-0.6010	0.999
		0.546	0.7417		
		0.625	0.6900		
	I	0.347	0.8999	-0.5877	0.995
		0.533	0.7825		
		0.610	0.7480		
	J	0.356	0.8628	-0.8967	0.999
		0.546	0.7294		
		0.625	0.6095		

Table S2. Hematocrit values, rivaroxaban relative recoveries, as well as the slopes and determination coefficients calculated for each whole blood sample (A thru J) in the investigation of the relationship between hematocrit, analyte concentration, and relative recoveries. The experiments were performed by spiking the analytes to each sample at a low, and at a high level.

Spiking Level	Sample	Hematocrit	Relative Recovery (%)	Slope	Determination Coefficient
Low	A	0.286	0.9984	-1.0798	0.910
		0.410	0.9660		
		0.607	0.6658		
	B	0.279	1.0162	-0.8403	1.000
		0.400	0.9092		
		0.593	0.7520		
	C	0.293	0.9937	-0.1993	0.701
		0.420	0.9284		
		0.622	0.9224		
	D	0.300	1.0019	-0.8469	0.860
		0.430	0.9992		
		0.637	0.7317		
	E	0.321	1.0004	-0.9812	0.934
		0.460	0.9519		
		0.681	0.6591		
	F	0.347	1.0630	-0.7624	0.998
		0.533	0.9143		
		0.610	0.8648		
	G	0.339	0.9790	-0.2445	0.933
		0.520	0.9473		
		0.596	0.9122		
	H	0.356	0.8823	0.3000	0.901
		0.546	0.9193		
		0.625	0.9694		
	I	0.347	1.0163	-0.0742	0.036

		0.533	0.9270		
		0.610	1.0207		
	J	0.356	0.9249	-0.2759	0.194
		0.546	0.7594		
		0.625	0.8865		
High	A	0.286	1.0108	-1.0106	0.888
		0.410	0.9932		
		0.607	0.7013		
	B	0.279	1.0175	-1.2514	0.832
		0.400	1.0304		
		0.593	0.6484		
	C	0.293	0.9969	-0.9717	0.989
		0.420	0.9052		
		0.622	0.6815		
	D	0.300	1.0277	-0.9340	0.996
		0.430	0.9259		
		0.637	0.7157		
	E	0.321	1.0135	-1.1524	0.977
		0.460	0.9120		
		0.681	0.6063		
	F	0.347	0.9955	-0.7541	0.545
		0.533	0.7202		
		0.610	0.8401		
	G	0.339	0.9257	-0.5656	0.949
		0.520	0.7982		
		0.596	0.7886		
	H	0.356	0.9202	-0.4875	0.914
		0.546	0.8576		
		0.625	0.7793		
	I	0.347	0.9635	-0.5971	0.998
		0.533	0.8573		
		0.610	0.8050		
	J	0.356	0.9592	-1.0469	0.959
		0.546	0.8036		
		0.625	0.6634		

Script S1. R script employed for performing Passong-Bablok regression and for the calculation of Lin's concordance correlation coefficient. The plotting algorithm included in this script had been published on the website https://rowannicholls.github.io/R/statistics/agreement/passing_bablok.html. There was no mention of the author, copyright, or any constraints of using the script. The rest of the script was written by one of the authors (G.B.K.).

```
# Passing-Bablok regression

library(mcr)

pbdata=read.csv(file.choose())

pb=mcreg(pbdata$lcms,pbdata$bcs,method.reg="PaBa") # first: x, second: y

cusum=MCResult.calcCUSUM(pb)

htest=cusum$max.cusum/sqrt(cusum$nNeg+1)


# Plotting

# Beginning of script published on https://rowannicholls.github.io/R/statistics/agree-
ment/passing_bablok.html


intercept_est <- pb@para[1]

intercept_lci <- pb@para[5]

intercept_uci <- pb@para[7]

gradient_est <- pb@para[2]

gradient_lci <- pb@para[6]

gradient_uci <- pb@para[8]

plot(pbdata$lcms, pbdata$bcs, xlab = "plasma, LC-MS/MS method (ng/mL)", ylab = "VAMS,
LC-MS/MS method (ng/mL)", cex.lab=0.2, cex.axis=2.25, type="n")


# Add legend

legend(2, 430,

  c(

    "Reference line",

    sprintf("%4.2fx + %4.2f", gradient_est, intercept_est),

    sprintf("Upper CI: %4.2fx + %4.2f", gradient_uci, intercept_uci),

    sprintf("Lower CI: %4.2fx + %4.2f", gradient_lci, intercept_lci)

  ),

  lty = c("dashed", "solid", "solid", "solid"),

  lwd = c(1, 2, 1, 1),

  col = c("black", "dodgerblue", "dodgerblue", "dodgerblue", 1),
```

```
  cex = 0.8
)

# Fill in colour between the confidence intervals
mylims <- par("usr")
x <- c(mylims[1], mylims[2], mylims[2], mylims[1])
y <- c(
  gradient_lci * x[1] + intercept_lci,
  gradient_lci * x[2] + intercept_lci,
  gradient_uci * x[2] + intercept_uci,
  gradient_uci * x[1] + intercept_uci
)
polygon(x, y, col = "grey")

# Reference line
abline(0, 1, lty = "dashed", lwd=2)

# Passing-Bablok regression lines
abline(intercept_est, gradient_est, col = "green", lwd = 2)
abline(intercept_lci, gradient_lci, col = "red")
abline(intercept_uci, gradient_uci, col = "red")

# Add data points
points(pbdata$lcms, pbdata$bcs, pch = 21, cex=1.5, col = "black", bg="black", xlim =
c(-10,550), ylim =  c(-10,550), xaxs = "i", yaxs = "i")

# End of script published on https://rowannicholls.github.io/R/statistics/agree-
ment/passing_bablok.html

# Lin's concordance correlation coefficient
library(DescTools)
lin=CCC(pbdata$lcms,pbdata$bcs)
```

Table S3. Results of the comparative apixaban experiments with real-life samples. Concentrations obtained in plasma employing LC-MS/MS and the functional anti-Xa chromogenic assay, as well as in dried whole blood volumetric absorptive microsamples evaluated by using dried plasma calibrators prepared on day 3, or dried whole blood calibrators prepared on day 0 of the seven-day samples processing cycle.

LC-MS/MS Assay in Plasma	Anti-Xa Assay in Plasma	VAMS Assay Using Dried Plasma Calibrators	VAMS Assay Using Dried Whole Blood Calibrators
6.8	13.0	2.3	2.3
106.3	115.2	104.7	83.7
7.5	13.0	13.5	10.9
75.5	83.4	94.5	78.2
79.2	83.5	68.0	68.2
57.1	59.0	48.2	47.3
60.2	57.1	49.4	48.5
344.3	357.7	291.2	314.4
432.2	506.0	459.4	501.2
9.3	15.3	9.5	9.1
170.3	176.0	180.2	164.9
82.3	99.0	78.6	73.6
28.6	32.8	18.6	19.6
61.3	67.1	48.1	50.4
151.7	167.8	131.8	138.7
91.1	93.9	70.1	74.0
89.4	182.7	148.0	156.2
0.9	1.0	2.5	2.6
103.8	219.0	84.9	89.8
87.6	170.0	150.6	162.4
35.9	98.9	48.0	48.4

Table S4. Results of the comparative rivaroxaban experiments with real-life samples. Concentrations obtained in plasma employing LC-MS/MS and the functional anti-Xa chromogenic assay, as well as in dried whole blood volumetric absorptive microsamples evaluated by using dried plasma calibrators prepared on day 3, or dried whole blood calibrators prepared on day 0 of the seven-day samples processing cycle.

LC-MS/MS Assay in Plasma	Anti-Xa Assay in Plasma	VAMS Assay Using Dried Plasma Calibrators	VAMS Assay Using Dried Whole Blood Calibrators
9.4	13.4	4.1	8.9
466.4	453.4	630.7	650.3
112.6	202.8	144.8	147.8
231.3	328.0	322.3	330.7
105.9	183.3	121.3	124.6
31.3	31.4	30.7	32.8
206.1	431.3	251.5	256.7
46.1	65.9	44.4	46.8
189.0	367.3	315.5	321.9
19.5	25.3	18.7	20.4
65.3	115.4	74.8	77.4
187.1	417.0	214.3	218.7
26.6	33.2	26.2	23.1
114.5	156.1	126.5	118.4

4.5	6.5	4.9	5.1
41.9	38.0	35.5	36.3
18.2	23.2	5.6	5.8
173.9	238.4	158.8	163.4
168.9	225.6	153.3	163.3
68.8	78.1	68.4	71.7
24.7	27.0	24.0	23.9
89.4	122.1	87.3	88.0
23.8	33.0	28.4	30.7
23.8	30.0	26.4	28.9
19.2	27.0	17.6	18.5
6.7	10.9	6.0	6.1
6.7	10.9	6.5	5.6
219.7	330.0	217.0	192.8
26.5	32.0	27.3	24.7
14.9	19.0	22.3	20.1
17.9	28.7	24.4	22.0
23.6	35.1	30.0	26.6
235.0	405.1	194.7	169.8
21.3	33.8	26.9	24.6
30.2	44.5	40.7	35.3
51.8	79.0	54.3	55.9
36.2	44.0	31.6	32.1
107.2	132.7	88.1	91.1
199.3	262.0	211.3	224.4
